# Mechanisms of Surfactin from *Bacillus subtilis* SF1 against *Fusarium foetens*: A Novel Pathogen Inducing Potato Wilt

**DOI:** 10.3390/jof9030367

**Published:** 2023-03-17

**Authors:** Lin Liu, Xiaofan Jin, Xiuhua Lu, Lizhong Guo, Peiwei Lu, Hao Yu, Beibei Lv

**Affiliations:** 1 College of Life Science, Shandong Province Key Laboratory of Applied Mycology, Qingdao Agricultural University, Qingdao 266109, China; 2 Bayer Crop Science China Co., Ltd., Hangzhou 310018, China; 3 Biotechnology Research Institute, Key Laboratory of Agricultural Genetics and Breeding, Shanghai Academy of Agricultural Sciences, Shanghai 201106, China

**Keywords:** biocontrol, *Fusarium* wilt, antifungal mechanism, cell membrane permeability, protein expression differential, DNA binding

## Abstract

*Fusarium* wilt is a severe and worldwide disease in potato cultivation. In this study, *Fusarium foetens* was first identified as the pathogen of potato wilt. *Bacillus subtilis* SF1 has the potential for controlling potato wilt induced by *F. foetens,* resulting in a mycelium growth inhibition of 52.50 ± 2.59% in vitro and a significant decrease in incidence rate by 45.56% in vivo. This research highlighted the antifungal activity of surfactin from *B. subtilis* SF1 and attempted to reveal the unknown antifungal mechanisms. Surfactin inhibited *F. foetens* mycelium growth beyond the concentration of 20 μg/μL. Surfactin-treated mycelium appeared to have morphological malformation. Surfactin enhanced reduced glutathione production and caused the increase in values of the extracellular fluids in OD260 and OD280. Surfactin induced differential protein expression and changed the genes’ transcription levels. Surfactin binds to fungal DNA via groove-binding mode, with a binding constant of *K_b_* 2.97 × 10^4^ M^−1^. Moreover, *B. subtilis* SF1 harbored genes encoding plant-promoting determinants, making potato seedlings grow vigorously. The results will help provide a comprehensive understanding of the mechanisms of surfactin against filamentous fungi and the application of surfactin-producing microbial in the biocontrol of plant pathogenic fungi.

## 1. Introduction

Potato (*Solanum tuberosum* L.) is the fourth largest crop, after rice, maize, and wheat. It ranks first as a non-cereal food crop for human consumption and has great potential for ensuring food security in developing countries [1]. About 1.3 billion people in China and India consume fresh potatoes as a staple food [2]. Diseases caused by fungi have always been a severe problem in potato cultivation [3]. *Fusarium* spp. are the most common soil-borne pathogens responsible for yield and commercial losses during potato production [4]. These pathogens affect potatoes at any growth stage by inducing *Fusarium* wilt (FW) on plants and *Fusarium* dry rot on tubers. FW is prevalent worldwide, leading to 30–78% yield losses in some parts of China [5]. FW is a vascular disease mainly caused by *F. oxysporum. Fusarium* spp., such as *F. solani*, *F. graminearum*, and *F. sambucinum*, have also been reported as FW pathogens [6,7,8].

Management strategies for potato *Fusarium* diseases have mainly focused on chemical fungicides, crop rotation, and breeding of resistant cultivars [4,9,10]. However, due to the rapid differentiation of pathogenicity, the appearance of resistant pathogenic strains, and the lack of superior cultivars with high levels of resistance, *Fusarium* diseases are difficult to control. A recent approach that has gained attention is the use of bacterial antagonists, such as members of the genus *Bacillus,* particularly *Bacillus subtilis,* which show the potential for controlling FW [11]. *B. subtilis* inhibits the growth of harmful fungal parasites within the rhizosphere and significantly increases plant growth [12]. Studies have established that non-ribosomally synthesized antibiotics, specifically, cyclic lipopeptides (LPs), are involved in most of the biocontrol mechanisms of different strains of *Bacillus* and play an important role in the control of fungal diseases [13]. Surfactin, a type of cyclic lipopeptide produced by *Bacillus* spp., possesses many biological activities, such as surfactant, antibacterial, antifungal, and antitumor activities [14]. Surfactin is well-known for its antibacterial action. Moreover, surfactin has received increasing attention for its antifungal activities, particularly for its inhibitory effects against phytopathogenic fungi belonging to the genera *Fusarium*, *Lasiodiplodia*, *Colletotrichum*, *Botryosphaeria*, *Aspergillus*, and *Penicillium* [15,16,17,18,19].

*Fusarium foetens* is a soil-borne plant pathogenic microorganism that was first reported to cause wilt in hybrid begonias in the Netherlands in 2000. These studies were rapidly followed by reports from Germany, England, and the USA in 2003, in France in 2007, and in Canada in 2010 [20,21,22,23]. *F. foetens* is relatively host-specific to begonia. However, since 2017, *F. foetens* has been reported as a pathogen of rooibos seedlings and Solanaceae crops, such as tomatoes, bell peppers, and cayenne peppers [24,25]. This fungus is morphologically difficult to distinguish from other members of the *F. oxysporum* species complex and other known *Fusarium* spp. Schroers et al. revealed that *F. foetens* can be identified by sequencing a portion of the nuclear translation elongation factor-1α (EF-1α) or β-tubulin genes and by randomly amplified polymorphic DNA analysis [21]. During an investigation of field potatoes in Laiyang city (120.71° E, 36.98° N), Shandong Province, China in 2021, our team found many wilted and dead potato plants in the fields. The disease had spread rapidly, more than 70% of the plants were infected, and all infected plants died within 2–3 weeks. Compared with the previous FW caused by *F. oxysporum* in this area, the disease had a faster onset, wider spread, and a higher mortality rate.

In this study, *F. foetens* was identified as the causal agent inducing potato wilt in Laiyang city, which is the first report of *F. foetens* infecting potatoes. The potential of *B. subtilis* SF1 in controlling potato wilt induced by *F. foetens* was assessed in vitro and in vivo. Surfactin was confirmed to be the key antifungal substance produced by *B. subtilis* SF1. The mechanisms of surfactin against *F. foetens* were studied systematically by investigating the effects of surfactin on mycelial growth, mycelial morphology, cell contents, protein expression, reduced glutathione (GSH) production, gene transcription levels, and genomic DNA of *F. foetens*. Although many studies have investigated the antifungal activities of surfactin, few have explored the underlying mechanisms, or conducted systematic research on the biocontrol of *F. foetens*. This study has practical significance for the rapid diagnosis, prevention, and control of FW induced by *F. foetens* on potatoes and other crops. The results will be helpful for a comprehensive understanding of the mechanisms of using surfactin against filamentous fungi and for utilizing surfactin-producing microbial agents in the biocontrol of plant pathogenic fungi.

## 2. Materials and Methods

### 2.1. Pathogen Isolation and Pathogenicity Tests

The symptomatic potato plants were collected from Laiyang, Shandong Province of China, in April 2021. The pathogen was isolated according to our previous publication [26]. After cleaning and sterilization, the stem base of the symptomatic potatoes was cut into pieces 1 cm long and 0.5 cm wide, placed on potato dextrose agar (PDA) Petri dishes, and cultured at 25 °C for 3–5 days. The obtained colonies were transferred onto a new plate repeatedly.

Three *F. foetens* isolates were selected randomly and conducted infection on 90 potato seedlings grown under greenhouse conditions for pathogenicity tests. Each isolate infected ten potato seedlings each time. The experiments were repeated three times. The seed tubers were planted in pots of peat and perlite (3:1 *v*/*v*). Two weeks later, The seedlings were transplanted into new pots with *F. foetens* spores (1 × 10^7^ CFU/mL) at a volume of 100 mL. The seedlings in pots with sterilized water were the negative control. All the seedlings were maintained in the greenhouse with 12 h light and 12 h dark per day at 25 °C for 30 days. The disease symptoms were investigated and the symptomatic to re-isolate the pathogen was collected.

### 2.2. Pathogen Identification

Multiple gene loci, internal transcribed spacer rDNA (ITS), EF-1α, and β-tubulin, were applied to the molecular identification of the pathogen causing potato wilt [21]. The ITS, EF-1α, and β-tubulin genes were amplified from the genomic DNA with PCR primers ITS1/ITS4, EF-1/EF-2, and T1/T22 [27,28], respectively. The amplified products were purified and sequenced. Then, the gene sequences were analyzed using BLAST in the NCBI database. The gene sequences of EF-1α and β-tubulin were combined to build a phylogenetic tree with Neighbor-Joining (NJ) in MEGA 10.0. The branch supports of NJ analysis were evaluated using a bootstrapping (BS) method of 1000 replicates.

The fungus grown on PDA was incubated at 25 °C in the dark for 10–14 days. The colony morphology of the fungus was observed using stereo microscope (M205C, Leica, Wetzlar, Germany). The morphology of conidia, phialide, and chlamydospore was observed with positive fluorescence microscope (CX21, Olympus, Tokyo, Japan) after staining the fungal structures with cotton blue.

### 2.3. The Antifungal Activity of B. subtilis SF1 against F. foetens In Vitro

*B. subtilis* SF1 (ACCC 19742) was purchased from the Agricultural Culture Collection of China. Illumina was used for the whole-genome sequencing of *B. subtilis* SF1 by oeBiotech (Shanghai, China). The whole genome shotgun project has been deposited at DDBJ/ENA/GenBank under the accession JANYMH000000000. The antifungal activity of *B. subtilis* SF1 against *F. foetens* was evaluated with a dual culture plate assay, as described in the previous study [29]. To investigate the antifungal activity of the metabolites produced by *B. subtilis* SF1, the culture filtrate of *B. subtilis* SF1 was prepared and mixed with water in different volumes to prepare the PDA medium. *F. foetens* plug (5 mm) was inoculated onto the plates containing different proportions of culture filtrate. The colony diameters of *F. foetens* were measured when the mycelium of control completely overspread the Petri dish surface. The growth inhibition for *F. foetens* was estimated using the formula: Growth inhibition% = ((D1 − D2)/D1) × 100%, where D1: Mean diameter of the colony for the control and D2: Mean diameter of the colony in presence of culture filtrate or cells of *B. subtilis* SF1. All the cultures were incubated at 25 °C.

The culture filtrate of *B. subtilis* SF1 was prepared following the procedures [30]: *B. subtilis* SF1 cells were inoculated into LB and cultured for 24 h. Then, the culture was transferred into the fermentation media consisting of glucose 40 g/L, beef extract 15 g/L, (NH_4_)_2_SO_4_ 4 g/L, KH_2_PO_4_ 14 g/L, 14 g Na_2_HPO_4_ g/L and MgSO_4_ 1.5 g/L. After being cultured for 40 h, the fermentation broth was centrifuged (4 °C, 6000× *g*), and the supernatant was collected and sterilized twice with a 0.22 μm millipore filter.

### 2.4. Effect of B. subtilis SF1 on Fusarium Wilt Incidence

The tests on the effect of *B. subtilis* SF1 on potato FW incidence were performed by the root-dip method previously described [31]. Wash roots of potato seedings, then dip into conidia suspension (1 × 10^7^ CFU/mL) of *F. foetens*. Transplant the infected seedlings into the pots supplemented with 100 mL of *B. subtilils* SF1 cell suspension (1 × 10^7^ cells/mL). Use sterilized water in place of *B. subtilis* SF1 culture in positive control. The seedlings without infection and *B. subtilis* SF1 inoculation were negative control. The disease incidence was assessed after 60 days using the following formulas: Incidence rate (%) = (No. of diseased plants/30) × 100%. To investigate the effect of *B. subtilis* SF1 on potato plant growth, the pots of healthy potato seedlings were inoculated with *B. subtilis* SF1 cells (100 mL, 1 × 10^7^ cells/mL). After culturing for 20 days, compare the seedlings treated with SF1 with the seedlings in negative control by monitoring the number of leaves and plant height. Ten potato seedlings were used in each experiment group each time. The experiments were repeated three times, and 120 potato seedlings were used. All the pots were put in the field under natural lighting. The average outdoor temperature was 15 and 22 °C at night and daytime, respectively.

### 2.5. Hydrolytic Enzymes Production

The protein-coding genes in the *B. subtilis* SF1 genome were annotated in the Carbohydrate-Active enZYmes database. The activities of cellulase, protease, β-glucanase, and chitinase were assessed by measuring the halo formation on agar plates supplemented with carboxymethyl cellulose, skim milk, barley flour, and colloidal chitin, respectively, in the basal medium LB [32,33]. The concentration of skim milk was 10%, and the concentration of other additives was 1%. The agar plates were cultured at 30 °C for 5 days. A clear halo zone around the colony indicated *B. subtilis* SF1 was positive for enzyme production.

### 2.6. Plant Growth Promoting-Determinants Produced by B. subtilis SF1

Based on the published gene sequences, the genes associated with plant promotion were blasted in the *B. subtilis* SF1 genome. The phosphate solubilization was confirmed by the appearance of a halo zone around the *B. subtilis* SF1 colony on agar after bromophenol blue staining [34]. Use the National Botanical Research Institute’s Phosphate growth medium (NBRIP) to examine inorganic phosphate solubilization. Replace the phosphorus source in the NBRIP with calcium phytate to assess organophosphates solubilization. *B. subtilis* SF1 was inoculated onto chromium azure S (CAS) medium, incubating for 5 days at 30 °C, to determine siderophores production by the appearance of a halo zone around the colony on CAS [35]. Indole-3-acetic acid (IAA) production was evaluated by the method of Bric et al. [36].

### 2.7. Extraction, Purification, and Identification of Surfactin Produced by B. subtilis SF1

The surfactin produced by *B. subtilis* SF1 was extracted using the acid precipitation method, as described previously [37]. The extracted surfactin was analyzed with the high-performance liquid chromatography (HPLC, 1100, Agilent, Santa Clara, CA, USA) equipped with a YMC C18 column (4.6 × 2.50 mm, 5 μm). The mobile phase consisted of 90% acetonitrile and 10% water (0.05% trifluoroacetic acid) with a flow rate of 0.8 mL per minute at 30 °C. The standard surfactin was purchased from Abison Company (Beijing, China).

The purification of surfactin was performed by preparative chromatography using the HPLC system (SCL-10A, Shimadzu, Tokyo, Japan) with a YMC C18 column (10.0 × 250 mm, 5 μm). The molecular weight of the surfactin was identified with LC-MS system (Orbitrap Fusion Lumos, Thermo Fisher, Waltham, MA, USA) equipped with a Kromasil C18 column (4.6 × 150 mm, 5 μm) and monitored at 210 nm as well as in positive-ion mode. The solvent gradient used acetonitrile and water at a flow rate of 3 mL/min with sample elution starting with 70% acetonitrile, followed by a linear gradient to 100% acetonitrile over 30 min.

### 2.8. Effect of Surfactin on the Growth and Morphology of F. foetens

The effect of surfactin on *F. foetens* growth was tested by the filter paper disk method [38]. The filter paper was dipped into 10 μL of surfactin at the concentration of 5, 10, 20, and 50 μg/μL, respectively, dried, and placed on a plate coated with 100 μL of 1 × 10^6^ CFU/mL of *F. foetens* spores. A filter paper sheet dipped into the sterilized water was the control. The plates were incubated at 25 °C for 40 days and observed for mycelium growth.

To investigate the effect of surfactin on *F. foetens* morphology, the *F. foetens* mycelium cultured for 5–7 days on PDA was picked onto a glass slide and added surfactin to a final concentration of 1 μg/μL to immerse the mycelium. The mycelium treated with sterile PBS was the control. The glass slides were placed on a moisturizing gauze in Petri dishes, incubating at 25 °C for 4 h. Then, the mycelium morphology was observed under positive fluorescence microscopy (Axio Scope A1, Leica, Wetzlar, Germany) and scanning electron microscopy (SEM, JSM-7500F, JEOL, Tokyo, Japan). The specimen preparation for SEM was according to a previous publication [17].

### 2.9. Effect of Surfactin on the Nucleic Acids and Proteins of F. foetens

*F. Foetens* spores (1 × 10^6^ CFU/mL) were added to PDB supplemented with 0.5 and 1 μg/μL of surfactin, respectively, and incubated at 25 °C for 1 to 4 h. Culture in PDB without surfactin was the control. At the end of the incubation, centrifuged at 5000× *g* for 5 min, then the supernatant was collected and measured at OD_260_ and OD_280_ levels to assess the leakage of nucleic acids and proteins from *F. foetens*.

To study the effect of surfactin on *F. foetens* protein expression, the PDB medium inoculated with 1 × 10^6^ CFU/mL spores was added to 0.5 μg/μL of surfactin and then incubated at 25 °C for 120 h. Culture in PDB without surfactin treatment was the control. After incubation, the mycelium was collected by centrifugation, and the total intracellular protein was extracted and separated by 12% sodium dodecyl sulfate-polyacrylamide gel electrophoresis (SDS-PAGE).

### 2.10. Effect of Surfactin on Transcription Level of F. foetens

The effect of surfactin on the mRNA levels was determined using quantitative PCR (qPCR). The *F. foetens* cells were cultured in PDB with/without surfactin (0.5 μg/μL) for 120 h as the above method in 2.9. After culturing, the total RNA of the collected mycelium was extracted with QIAGEN RNeasy Mini Kit (QIAGEN, Hilden, Germany). cDNA was synthesized from purified total RNA using the QuantiTect reverse transcription kit (QIAGEN, Hilden, Germany). qPCR was performed using PrimeScriptTM RT reagent Kit with gDNA Eraser (Takara, Osaka, Japan). β-actin was selected as the reference gene to adjust the veracity of the qPCR assay. Primers were designed by Tsingke Biotechnology Co., Ltd., Beijing, China. Three parallel measurements were performed for every sample in a LightCycler 96 real-time PCR instrument (CFX96 touch, Bio, Washington, DC, USA). Relative transcription levels were presented using the 2^−ΔΔ^^CT^ method.

### 2.11. Effect of Surfactin on GSH Production of F. foetens

To investigate the effect of surfactin on GSH production in *F. foetens*, the *F. foetens* spores (1 × 10^6^ CFU/mL) were inoculated in PDB without and with surfactin (0.5, 1, and 2 μg/μL, respectively). After incubation at 25 °C for 120 h, the mycelium was collected and mixed with 10% trifluoroacetic acid at a volume ratio of 1:1. The mixture was centrifuged at 5000× *g* for 5 min, then add 0.1 M potassium phosphate buffer into the supernatant. Ellman’s reagent was used to measure the absorbance value at 412 nm using a spectrophotometer after 5 min.

### 2.12. DNA Binding Assay

The interaction between surfactin and the genomic DNA of *F. foetens* was detected by gel retardation experiments, as described previously [39]. The genomic DNA was extracted, and its purity was tested by OD_260_/OD_280_ > 1.8. *F. foetens* DNA (ff-DNA) was treated with surfactin at concentrations of 0.05, 0.1, 0.5, and 1 μg/μL, respectively, and incubated for 1 h at room temperature, then subjected to electrophoresis on a 1% agarose gel and photographed under UV observation.

The binding mode of surfactin with ff-DNA was confirmed using UV-visible and fluorescence spectroscopy. The UV-visible spectroscopy analysis referred to a variation of the previous method [40]. The concentration of ff-DNA (30 μg/mL) was kept constant in 0.01 M PBS (pH 7.2) while gradually increasing the concentrations of surfactin by dropping the surfactin solution (1 μg/μL). It was equilibrated for 10 min after each drop, then the absorption spectrum of ff-DNA was measured in the range of 200 to 320 nm using an UV-visible spectrophotometer (Cary60, Agilent, Santa Clara, CA, USA). The fluorescence spectroscopy analysis was performed, as previously described [41]. Surfactin is a non-fluorescent compound, and ethidium bromide (EB) was used as a fluorescent probe to examine the interaction between surfactin and ff-DNA. The concentration of ff-DNA (30 μg/mL) was kept constant in 0.01 M PBS (pH 7.2), equilibrated for 10 min after EB (1.59 × 10^−5^ M) addition, and then equilibrated for 20 min after each successive drop of surfactin solution (1 μg/μL). The fluorescence emission spectra in the range of 520 to 620 nm were scanned at room temperature using a fluorescence spectrophotometer (F4600, Hitachi, Tokyo, Japan) with a fixed excitation wavelength of 485 nm, an incident slit of 5 nm and an exit slit of 5 nm.

### 2.13. Statistical Analysis

The diameters of the colonies were expressed as mean values ± SD. All the assays were repeated in at least three separate experiments. Data were subjected to analysis of variance using SPSS version 20.0 for windows. Mean values were compared using Duncan’s multiple range test at the 5% (*p* < 0.05) level of significance.

## 3. Results

### 3.1. Molecular and Morphological Characteristics of the F. foetens Pathogen

#### 3.1.1. Isolation of the Pathogen and Molecular Identification

**Figure 1:**
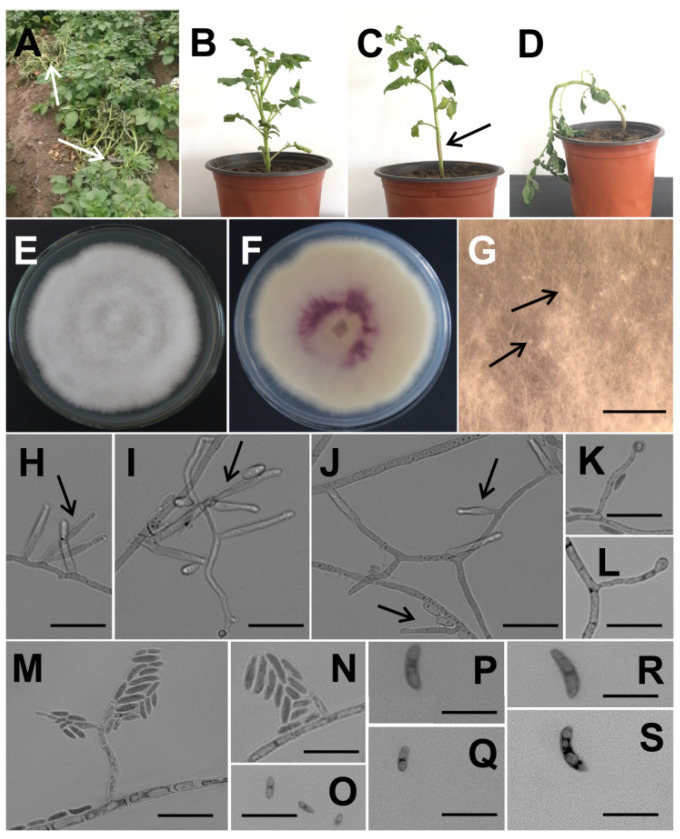
Morphological characteristics of the mycelium and spores of *F. foetens* and its pathogenicity. (**A**) The potato plants with wilt symptoms in the field. (**B**) Negative control: the potato seedlings in pots inoculated with sterilized water and cultivated in the greenhouse. (**C**,**D**) the potato seedings after infection with *F. foetens* for 4 to 6 and 9 days, respectively. (**E**,**F**) The front and the reverse colony of the *F. foetens* on PDA, cultured at 25 °C and for 7 days. (**G**) The dotted appearance formed on aerial mycelia, the scale bar is 5 μm. (**H**–**J**) The phialides of the mycelium, the scale bar is 20 μm. (**K**,**L**), The chlamydospore, the scale bar is 20 μm. (**M**–**S**) The microconidia, the scale bar is 20 μm.

Twenty potato plants with typical wilt symptoms were collected from Laiyang city, China (Figure 1A). A total of 99 fungi were isolated from the stem base of these diseased plants. The ITS fragments of these fungi were amplified and sequenced. The ITS sequence alignment results in the NCBI database showed that these fungi belonged to the genera *Fusarium*, *Alternaria*, and *Colletotrichum*, accounting for 75.76% (75), 18.19% (18) and 6.06% (6), respectively. Subsequently, partial sequences of EF-1α and β-tubulin were used to further identify 75 *Fusarium* strains at the species level. Fifty-three *Fusarium* isolates shared identical ITS (OM370930, NCBI), EF-1α (OM370932, NCBI), and β-tubulin (OM370931, NCBI) sequences, which showed 100% identity to the corresponding gene sequences of *F. foetens* CBS 110,286 (ITS for NR_159865.1, EF-1α for MT011001.1, β-tubulin for MT011049.1 in NCBI). A phylogenetic tree was constructed using the neighbor-joining method with the combination of the EF-1α and β-tubulin gene sequences from 10 *Fusarium* strains (Supplementary Figure S1). The *F. foetens* isolate was grouped with *F. foetens* CBS 110286 and was closely related to *F. oxysporum*.

#### 3.1.2. Morphological Characteristics of *F. foetens*

The colony morphology of *F. foetens* is shown in Figure 1E,F. The aerial mycelia in the front colony formed thick white tufts evenly covering the entire Petri dish, whereas the reverse colony appeared purplish-red in the center. The colony diameter was 33.8 ± 1.6 mm on PDA after 4 days, and 57.0 ± 1.9 mm after 7 days. The average radial growth rate was 8.2 ± 0.3 mm/day. Conspicuous aerial mycelia gave older colonies a dotted appearance (Figure 1G), which appeared to be stromata-supporting sporodochia [21]. However, no sporodochia occurred until 28 days of culture. Aerial mycelia bore solitary monophialides, occasionally polyphialides (Figure 1H–J). The phialides were either cylindrical or slightly tapered toward the tip or narrowly flask-shaped, with the widest point in the middle (Figure 1J). The chlamydospores were globose to subglobose, rare, mostly terminal, and smooth (Figure 1K,L). Microconidia that formed laterally from hyphae of the aerial mycelium were 0 to 3 septate and fusiform to slightly curved (Figure 1M–S). It is worth noting that *F. foetens* produced a pungent distinct odor.

#### 3.1.3. Pathogenicity Test

Three representative *F. foetens* isolates were selected randomly and inoculated into potato seedlings pots cultivated in the greenhouse. After 20 days of cultivation in the greenhouse, the seedlings in the negative control group did not exhibit any wilting symptoms (Figure 1B). The tested fungi developed wilting symptoms and were re-isolated from the infected plants, indicating that they were the pathogen causing the potato wilt. After inoculating with *F. foetens* on days 4–6, the potato plant stems near the roots appeared purplish red, this color gradually spread to the top of the stem, and the lower leaves began to wilt (Figure 1C). On day 9, the plant tilted showing a damping-off trend (Figure 1D). As the disease worsened, the plant damped and died after 2–3 weeks. Therefore, *F. foetens* was identified as the pathogen of potato FW, and this fungus was discovered to be responsible for potato wilt for the first time.

### 3.2. Evaluation of the Antifungal and Plant Growth-Promoting Properties of B. subtilis SF1

#### 3.2.1. The Antifungal Activity of *B. subtilis* SF1 against *F. foetens* In Vitro

**Figure 2:**
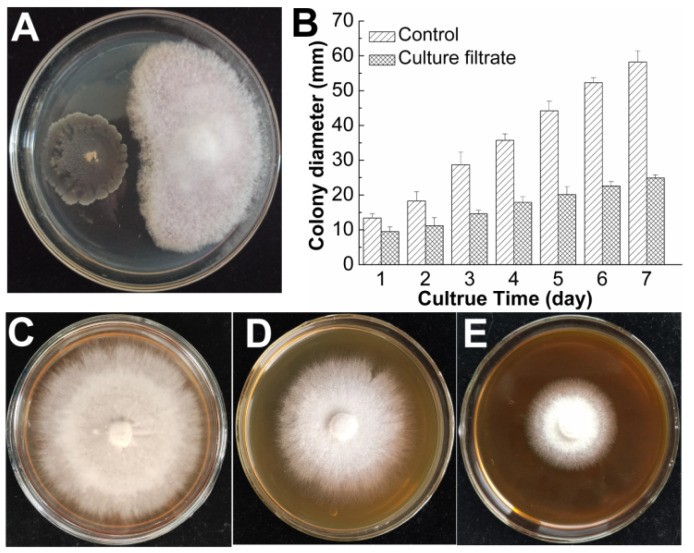
The antifungal activity of *B. subtilis* SF1 against *F. foetens* in vitro. (**A**) The confrontation assay of *B. subtilis* SF1 and *F. foetens* on PDA. (**B**) The colony diameter of *F. foetens* on PDA with and without *B. subtilis* SF1 culture filtrate. (**C**–**E**) The colony of *F. foetens* on PDA supplemented with different volumes of culture filtrate, the volume ratio of culture filtrate to water was 0:5, 2:3, and 5:0, respectively.

The antifungal activity of *B. subtilis* SF1 against *F. foetens* was evaluated in vitro. As shown in Figure 2A, *F. foetens* was significantly inhibited by *B. subtilis* SF1 in the confrontation assay, revealing 52.50 ± 2.59% inhibited growth. To investigate the antifungal activity of the metabolites produced by *B. subtilis* SF1, the *F. foetens* spores were inoculated onto PDA mixed with different volumes of the SF1 culture filtrate. Figure 2C–E shows that the antifungal activity against *F. foetens* increased with an increase in the proportion of supplemented culture filtrate. When the volume ratios of the culture filtrate to water in the PDA medium were 3:2 and 5:0, growth rates were inhibited by 25.32 ± 1.96% and 55.76 ± 2.46%, respectively. Based on the *F. foetens* colony diameters in Figure 2B, the average mycelial growth rates were 3.6 ± 0.3 and 8.3 ± 0.3 mm/day on PDA with and without the *B. subtilis* SF1 culture filtrate, respectively. These results suggest that *B. subtilis* SF1 suppressed *F. foetens* with antifungal metabolites.

#### 3.2.2. Effect of *B. subtilis* SF1 on FW Incidence In Vivo

The efficacy of *B. subtilis* SF1 against potato FW induced by *F. foetens* was evaluated in vivo. The negative control seedlings did not exhibit any symptoms. The positive control seedlings (treated with *F. foetens*) presented 100% morbidity. Inoculating the *B. subtilis* SF1 spore suspension after infection resulted in a lower incidence rate of 54.44 ± 2.31%, which decreased by 45.56% compared to the positive control (Supplementary Figure S2). The healthy potato plants treated with *B. subtilis* SF1 spores grew better than the potato plants in the negative control, as they were taller and had more leaves (Supplementary Figure S2). These results indicate that *B. subtilis* SF1 alleviated the incidence of potato FW caused by *F. foetens* in vivo and promoted potato growth.

#### 3.2.3. Production of Hydrolytic Enzymes

**Figure 3:**
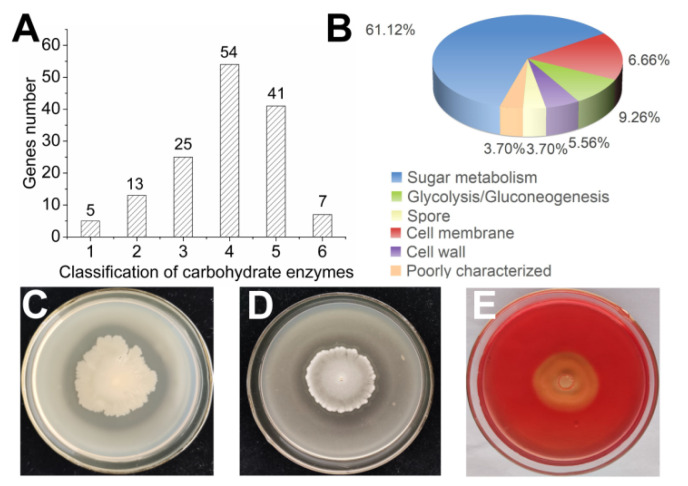
The classification and activities of hydrolytic enzymes of *B. subtilis* SF1. (**A**) The classification of carbohydrate enzymes in *B. subtilis* SF1 genome. (**B**) The function classification of the glycoside hydrolases in the genome of *B. subtilis* SF1. (**C**–**E**) The activities of protease, β-glucanase, and cellulase on the agar plate, respectively.

In total, 145 genes were annotated in the *B. subtilis* SF1 genome in the Carbohydrate-Active enZYmes database. Among the annotated genes, 54 genes encoded glycoside hydrolases, and most of these (61.12%) were involved in sugar metabolism (Figure 3A,B). Cellulase, β-glucanase, chitinase, and protease are fungal cell wall degradation-associated enzymes. Hydrolase assays on agar plates indicated that *B. subtilis* SF1 secreted protease, glucanase, and cellulase (Figure 3C–E). The undetected activity of chitinase on agar (data not shown) agrees with the result that no chitinase-encoding genes exist in the genome. The genes encoding beta-glucanase, endo-1,4-beta-glucanase, and extracellular protease in the genome of *B. subtilis* SF1 are shown in the Supplementary Table S1. To inactivate the heat-sensitive antifungal components in the *B. subtilis* SF1 metabolites, the *B. subtilis* SF1 culture filtrate was heated at 100 °C for 10 min, mixed with PDA to prepare the medium, and inoculated with *F. foetens*. Compared to the unheated sample (57.24 ± 2.37%), the inhibition of growth against *F. foetens* decreased (45.37 ± 2.07%) by heat treatment (Supplementary Figure S3). The remaining inhibited growth indicated that the vital antifungal components in the *B. subtilis* SF1 culture filtrate were heat-insensitive. Therefore, hydrolytic enzymes were not the antifungal ingredients that affected *F. foetens* in *B. subtilis* SF1.

#### 3.2.4. Plant Growth-Promoting Determinants Produced by *B. subtilis* SF1

**Figure 4:**
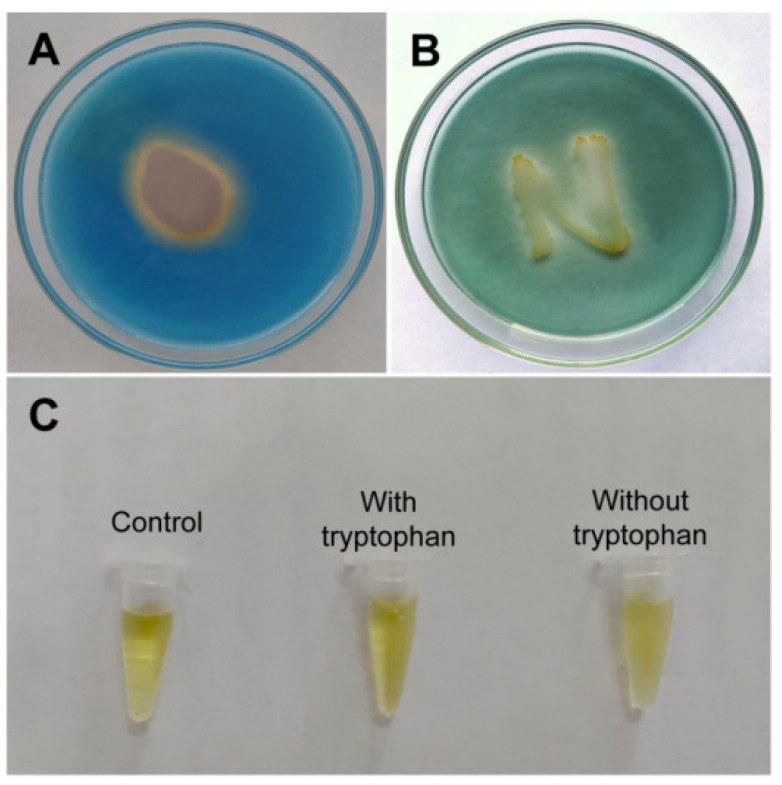
The plant growth-promoting determinants produced by *B. subtilis* SF1. (**A**) The siderophores production on CAS agar. (**B**) The organophosphorus solubilization assay on NBRIP. (**C**) The IAA production in LB with or without Tryptophan.

The genes involved in plant growth-promoting were summarized based on the genome analysis of *B. subtlis* SF1. As shown in Supplementary Table S2, *B. subtilis* SF1 contained a series of genes associated with root colonization, biofilm formation, swarming motility, and elicitation of plant basal defense. A clear halo zone was observed around the colony on CAS agar, indicating that *B. subtilis* SF1 produced siderophores (Figure 4A). A hydrolysis halo appeared around the *B. subtilis* SF1 colony on the organophosphate agar plate (Figure 4B), indicating that *B. subtilis* SF1 degrades organophosphates. No solubilization of inorganic phosphate was detected on the NBRIP plate (data not shown). No IAA was produced by *B. subtilis* SF1 as no color change occurred in the samples with or without tryptophan (Figure 4C), which was consistent with the absence of IAA synthesis-related genes in the *B. subtilis* SF1 genome. *B. subtilis* SF1 synthesized metabolites to enhance plant growth, illustrating why the potato seedlings treated with *B. subtilis* SF1 grew more vigorously than the negative control seedlings.

### 3.3. Purification, Identification and Antifungal Mechanisms of the Surfactin from B. subtilis SF1

#### 3.3.1. Purification and Identification of the Surfactin Produced by *B. subtilis* SF1

**Figure 5:**
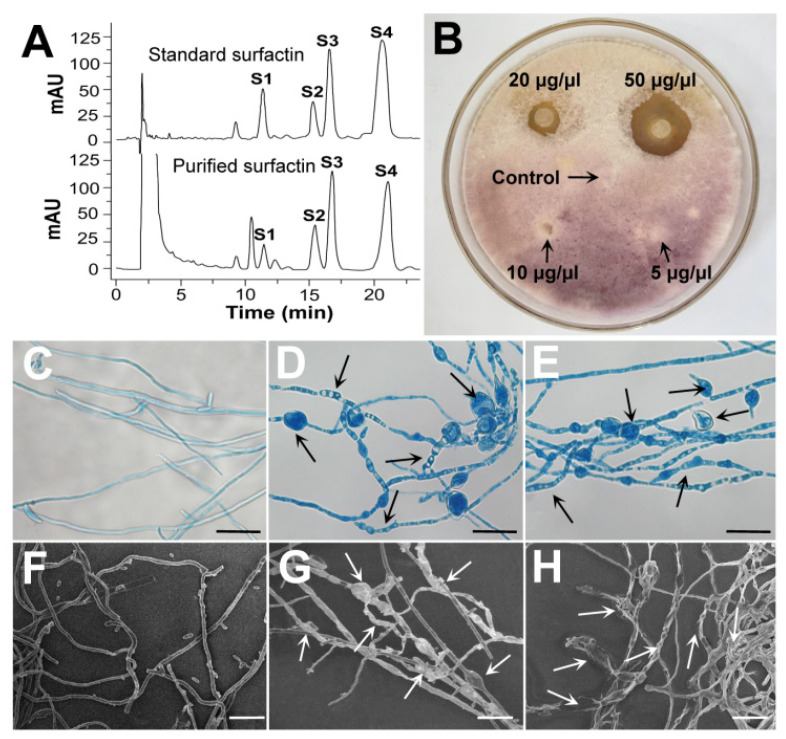
The HPLC fingerprint spectrum of surfactin produced by *B. subtilis* SF1 and its effects on the mycelium growth and morphology of *F. foetens*. (**A**) HPLC analysis of standard surfactin and the purified surfactin from *B. subtilis* SF1. (**B**) The antifungal activity of surfactin against *F. feotens* in vitro. (**C**,**F**) *F. foetens* mycelium without surfactin treatment under positive fluorescent microscopy and SEM. (**D**,**E**) Surfactin-treated *F. foetens* mycelium under positive fluorescent microscopy. (**G**,**H**) Surfactin-treated *F. foetens* mycelium under SEM.

The secondary metabolites produced by *B. subtilis* SF1 were predicted with the bacterial version of AntiSMASH (https://antismash.secondarymetabolites.org/, accessed on 22 April 2022). As shown in Supplementary Table S3, surfactin and pilpastatin possessed antifungal activity based on previous studies. The surfactin produced by *B. subtilis* SF1 was extracted and analyzed by HPLC. Crude surfactin produced four peaks in the HPLC fingerprint spectrum with the same retention times as the four surfactin standard peaks (Figure 5A). Crude surfactin was purified by preparative HPLC. The molecular mass of the purified surfactin was measured by liquid chromatography-mass spectroscopy (LC-MS). The four tested surfactin homologs produced main peaks at m/z 1030 (S1), 1044 (S2), 1044 (S3), and 1058 (S4), corresponding to molecular masses of 1007 (S1; C13), 1021 (S2 and S3; C14), and 1035 (S4; C15) Da, respectively (Supplementary Figure S4). S2 and S3 were isomers with the same molecular weight of 1021 Da. The relative contents of S1, S2, S3, and S4 in purified surfactin were 16.67, 19.51, 20.23, and 42.89%, respectively.

#### 3.3.2. Effect of Surfactin on the Growth and Morphology of *F. foetens*

The effect of surfactin on the growth of *F. foetens* was evaluated using the filter disk method (Figure 5B). The *F. foetens* mycelia initially spread over the filter paper as if surfactin had no inhibitory effect on the growth of *F. foetens*. After the 30-day incubation, the mycelia on/around the filter paper appeared to have significantly collapsed. The mycelia around the filter paper gradually began to dissolve and almost disappeared, indicating a clear circle of inhibition. Surfactin effectively inhibited the growth of *F. foetens* at a concentration of 20 μg/μL. Higher concentrations resulted in larger zones of inhibition. The antifungal effect of surfactin was significant but was slow and persistent.

To investigate the effect of surfactin on the *F. foetens* mycelial morphology, 1 μg/μL surfactin was used to treat the mycelia. The results of SEM and fluorescence microscopy are shown in Figure 5C–H. The mycelia of the control had smooth edges and uniform thickness (Figure 5C–F), while the surfactin-treated mycelia appeared to have an uneven, broken, and wrinkled surface (Figure 5D,E,G,H). The most significant morphological changes to the *F. foetens* mycelium caused by surfactin were abnormal vacuolation and swelling.

#### 3.3.3. Effect of Surfactin on *F. foetens* Membrane Integrity

**Figure 6:**
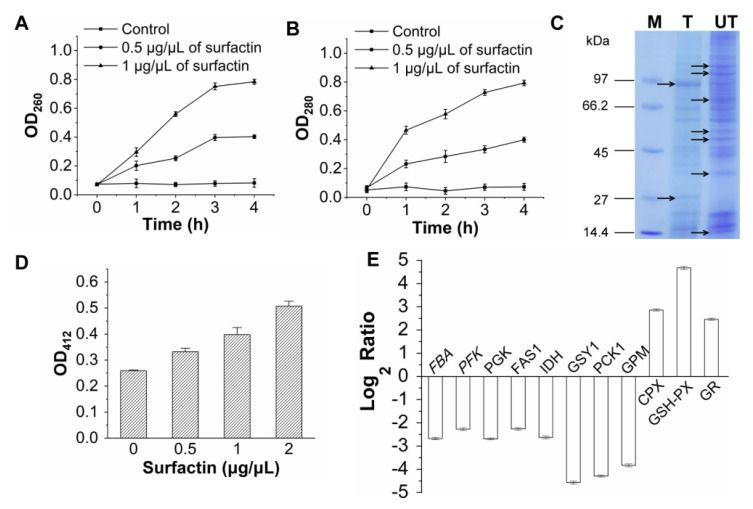
The effects of surfactin on the proteins, nucleic acids, GSH production, and gene transcription levels of *F. foetens*. (**A**) The effect of surfactin on the extracellular OD_260_. (**B**) The effect of surfactin on the extracellular DO_280_. (**C**) The effect of surfactin on the protein expression. (**D**) The effect of surfactin on GSH production. (**E**) The transcription levels of genes in *F. foetens* under surfactin treatment. *FBA* encodes fructose-bisphosphate aldolase; *PFK* encodes phosphofructokinase; *PGK* encodes phosphoglycerate kinase; *FAS1* encodes fatty acid synthase subunit beta; *IDH* encodes isocitrate dehydrogenase; *GSY1* encodes glycogen synthase; *PCK1* encodes phosphoenolpyruvate carboxykinase; *GPM* encodes phosphoglycerate mutase; *CPX* encodes catalase-peroxidase; *GSH*-*PX* encodes glutathione peroxidase; *GR* encodes glutathione reductase.

The effect of surfactin on *F. foetens* membrane integrity was reflected by the release of nucleic acids and proteins. *F. foetens* was cultured in PDB supplemented with different concentrations of surfactin, and the OD_260_ and OD_280_ values of the extracellular fluid were assessed. As shown in Figure 6A,B, the OD_260_ values increased with increasing surfactin concentration, and a similar profile was observed for OD_280_. These results demonstrated that nucleic acids and proteins were rapidly released into the external fungal body after the surfactin treatment. The leakage of nucleic acids and proteins suggested that the surfactin produced by *B. subtilis* SF1 damaged *F. foetens* membrane integrity.

#### 3.3.4. Effect of Surfactin on *F. foetens* Protein Expression

To determine the effect of surfactin on protein expression by *F. foetens*, the total intracellular proteins of *F. foetens* were collected and analyzed by SDS-PAGE after exposure to surfactin (0.5 μg/μL) for 120 h. As shown in Figure 6C, surfactin decreased the densities of some of the protein bands, and many of the bands were almost undetectable. The presence of surfactin also resulted in the appearance of several protein bands with significantly different molecular weights. These results suggest that surfactin produced by *B. subtilis* SF1 induced differential protein expression in *F. foetens*.

#### 3.3.5. Effect of Surfactin on GSH Production by *F. foetens*

GSH is an important intracellular non-enzymatic antioxidant. To determine the effect of surfactin on *F. foetens* intracellular GSH content, an *F. foetens* culture was treated with different concentrations of surfactin for 120 h. As shown in Figure 6D, the surfactin treatment resulted in increased GSH production, and GSH content increased with increasing concentrations of surfactin.

#### 3.3.6. Effect of Surfactin on *F. foetens* Transcription Levels

The transcription levels of some genes related to basal metabolism and antioxidation were determined to investigate the effect of surfactin on various metabolic pathways. As shown in Figure 6E, the transcription levels of genes, such as *FBA*, *PFK, *and *PGK* in glycolysis, *IDH* in the TCA pathway, *GSY1* in glycogen metabolism, *PCK1* and *GPM* in gluconeogenesis, and *FAS*1 in fatty acid metabolic processes, were downregulated. The gene expression levels of *CPX*, *GSH*-*PX*, and *GR* were upregulated.

#### 3.3.7. Interaction between Surfactin and ff-DNA by Electrophoresis, UV-Visible Spectroscopy and Fluorescence Spectroscopy

**Figure 7:**
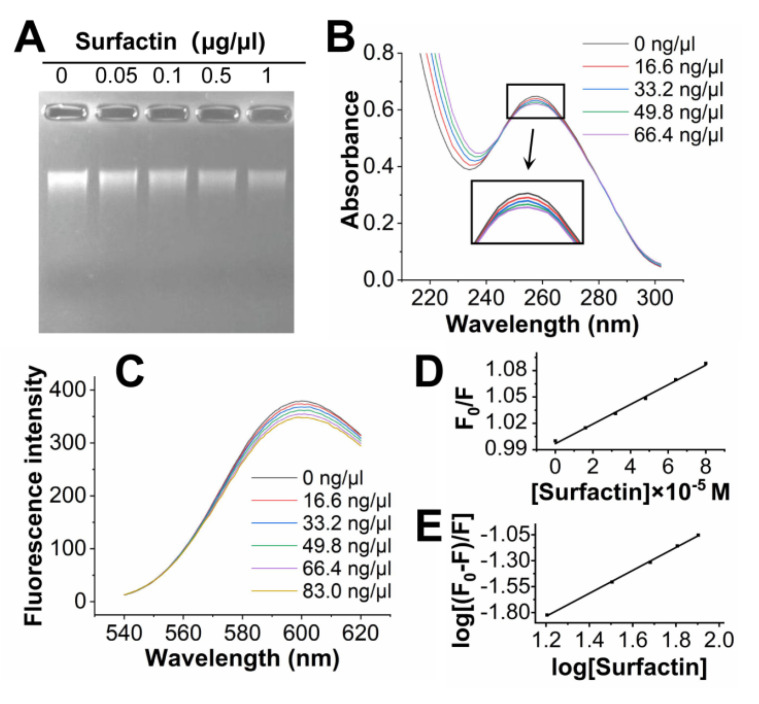
The interaction between surfactin and the genomic DNA of *F. foetens*. (**A**) Gel retardation assay after surfactin mixed with the DNA. (**B**) UV-Visible absorption spectra of *F. foetens* DNA with increasing concentrations of surfactin in PBS buffer (pH = 7.2). (**C**) The effect of surfactin on the fluorescence emission of ff-DNA-EB complex (λ_ex_ = 485 nm). (**D**) The Stern-Volmer plot of surfactin quenching effect on ff-DNA-EB complex fluorescence at room temperature. (**E**) The double-log plot of surfactin quenching effect on ff-DNA-EB complex fluorescence at room temperature.

ff-DNA was treated with different concentrations of surfactin and analyzed by electrophoresis. As shown in Figure 7A, the bands dimmed as the surfactin concentration was increased, the bands dimmed. DNA bound by surfactin was not displayed on the agarose gel, the results indicate that surfactin bound to ff-DNA.

Intercalations, grooves, and electrostatic interactions are non-covalent binding modes by which small molecules interact with DNA [42]. The UV-visible spectra of ff-DNA showed a slight decrease at 260 nm and no redshift (Figure 7B). The lack of a redshift indicates that the binding mode was not intercalation [43]. The small hypochromic effect indicates that the binding mode of surfactin to DNA might be groove or electrostatic interaction [44].

Because surfactin does not fluoresce, the binding mode of ff-DNA with surfactin was further confirmed by fluorescent EB-competitive binding. EB is a typical DNA-intercalation marker [45]. As shown in Figure 7C, the intensity of the emission band at 600 nm of the EB-DNA system decreased with increasing concentrations of surfactin when the EB-DNA solution was excited at 485 nm. Fluorescence quenching was due to the replacement of EB from the complex (EB-DNA) or when a new complex (EB-DNA-surfactin) formed. The fluorescence quenching constant was determined by the Stern-Volmer equation: *F*_0_/*F* = 1 + *K_q_τ*_0_ [Surfactin] = 1 + *K_SV_* [Surfactin], where *F*_0_ and *F* indicate the fluorescence intensities without and with the quencher surfactin, respectively, [Surfactin] is the concentration of surfactin, *τ*_0_ is the average fluorescence lifetime of the molecule and its value is 10^−8^ s. *K_SV_* is the Stern–Volmer quenching constant, which was obtained from the slope of *F*_0_/*F* versus [surfactin] plot (Figure 7D). Based on the calculation, the quenching rate constant, *K_q_*, was 1.07 × 10^11^ L/mol·s, which is greater than the maximum diffusion collision quenching rate constant of quenching agents on biomolecules (2 × 10^10^ L/mol·s). These results confirm that the quenching of EB-DNA fluorescence was initiated by the formation of a complex between surfactin and EB-DNA through a static mechanism rather than dynamic collision.

The binding constant (*K_b_*), as well as the binding sites (*n*) for quenching interactions, were determined using the fluorimetric data from the equation: log(*F*_0_ − *F*)/*F* = log*K_b_* + *n* log[Surfactin]. The *K_b_* and *n* values were calculated using the plot of log [(*F*_0_ − *F*)/*F*] versus log[Surfactin] (Figure 7E). The binding constant (*K_b_*) values of the classic intercalating agents EB (2.6 × 10^6^ M^−1^) and acridine orange (4.0 × 10^5^ M^−1^) are 10^5^ to 10^6^ M^−1^ [43]. In our experiment, the *K_b_* value of the surfactin was 2.97 × 10^4^ M^−1^, which was lower than that expected for an intercalating compound but was consistent with one that undergoes groove binding [40]. Moreover, only molecules with planar aromatic rings are involved in intercalation between DNA base pairs. The non-planar nature of the compounds also shows the non-intercalative binding with DNA [46,47]. The binding mode of ff-DNA and surfactin was electrostatic interaction or groove. Electrostatic binding occurs due to the interaction between the negatively charged phosphate backbone of DNA and the positively charged ends of small molecules [42]. Surfactin has a net negative charge [48]. Thus, all of these results support that surfactin undergoes groove binding to ff-DNA.

## 4. Discussion

FW induced by *Fusarium* is a severe disease that occurs in potato cultivation worldwide. It is difficult to prevent and control FW by selecting resistant varieties or using chemical agents [49]. *B. subtilis* spp. have been used to control FW on crops, such as cucumber, pepper, tomato, and muskmelon [50,51,52,53]. In this study, *F. foetens* was identified as the cause of potato FW. However, systematic studies on preventing *F. foetens* are unavailable. Surfactin produced by *B. subtilis* SF1 exhibited significant inhibitory activity against *F. foetens*, and the underlying antifungal mechanisms of surfactin were revealed.

*F. foetens* closely resembles *F. oxysporum* [54]. The colony and spore morphology of *F. foetens* are very similar to those of *F. oxysporum*. A pungent colony odor distinguished the fungus from *F. oxysporum*. The phylogenetic analysis indicated that *F. foetens* was closely related to *F. oxysporum. F. oxysporum* is well-known to cause FW during potato production. The closest relative provided some evidence for their similar morphology and the ability to infect potatoes to induce FW.

*B. subtilis* SF1 exhibited significant inhibitory activity against *F. foetens* in the in vitro assay. Growth inhibition of 55.76 ± 2.46% was observed when adding the *B. subtilis* SF1 culture filtrate to PDA, indicating that the antifungal activity was attributed to metabolites produced by *B. subtilis* SF1. Extracellular enzymes produced by *Bacillus*, such as chitinase, protease, β-glucanase, and cellulose, affect the growth and morphology of fungi [55,56]. These enzymes degrade the hyphal cell wall, change its permeability, and cause the hyphae to swell [57,58,59]. The hydrolytic enzymes perhaps were not the key antifungal ingredients produced by *B. subtilis* SF1, due to their lower activities on agar as well as the absence of chitinase-coding genes in the SF1 genome. The growth inhibition was still significant (45.37%) even after the *B. subtilis* SF1 culture filtrate was heated at 100 °C for 10 min, indicating that key antifungal components in *B. subtilis* SF1 were heat-resistant. These results suggest that hydrolytic enzymes made a limited contribution to the antifungal activity of *B. subtilis* SF1.

The secondary metabolites of *B. subtilis* SF1 were predicted with AntiSMASH. The presence and antifungal activity of surfactin was confirmed by HPLC and the filter paper disk method, respectively. Interestingly, the inhibiting effect of surfactin was slow and persistent. The *F. foetens* mycelia initially spread and grew on the filter paper. However, a clear inhibition zone became apparent around the filter paper disk after 30 days of exposure to surfactin. The antifungal characteristics of surfactin were significantly different from fengycin (another lipopeptide produced by *Bacillus*) and carbendazol, which directly inhibit the growth of fungus and form a clear circle of inhibition [39,60]. Various lipopeptide antibiotics produced by *B. subtilis* are highly stable at high temperatures, with more than 90% of its activity being retained even after samples had been held at 100 °C for 2 h [61,62]. The thermal resistance of surfactin is consistent with the result that heating did not destroy the activity of the antifungal substances in *B. subtilis* SF1. Thus, surfactin may play a key role in the activities of *B. subtilis* SF1 against *F. foetens*.

Based on the in vitro antifungal assays with the filter paper disk, surfactin produced by *B. subtilis* SF1 acted on the growing mycelia and eventually led to the death and lysis of the *F. foetens* mycelia. What are the underlying mechanisms used by surfactin to inhibit *F. foetens*? Surfactin induces ion-conducting pores in artificial lipid membranes and alters membrane permeabilization [63,64]. The leakage of nucleic acids and proteins of *F. foetens* after exposure to surfactin confirmed that surfactin damaged the membrane integrity of *F. foetens.* In a previous study, surfactin [ΔLeu6] (lacking amino acid Leu-6) possessed significant antifungal activity against *F. moniliforme* and induced leakage of nucleic acids and proteins [17]. On the other hand, surfactin changes mycelial morphology. The surfactin-treated mycelium appeared to have an uneven, broken, and wrinkled surface, with swelling and vacuolations. Similar morphological abnormalities were observed when *B. subtilis* IBFCBF-4 was co-cultured with *F. oxysporum* [65]. Hu et al. reported that fengycin changes the permeability of the cell membrane and causes *F. moniliforme* hyphae to appear vacuolated [66]. Surfactin may dehydrate and interact with the phospholipid acyl chains, resulting in considerable membrane fluidization [64]. Surfactin produced by *B. subtilis* SF1 changed the membrane permeabilization of *F. foetens* cells, inducing abnormal mycelial morphology and lysis of intracellular substances.

Can surfactin inhibit the fungus via other mechanisms? Surfactin is involved in the interaction between *B. subtilis* and *Aspergillus niger*, resulting in altered metabolism in the bacterium and the fungus [67]. Several new protein bands and a significant fading of the original protein bands on SDS-PAGE demonstrated that surfactin caused differential protein expression in *F. foetens*. Surfactin extracted from *Brevibacillus brevis* KN8(2) alters the protein expression of *F. moniliforme on* SDS-PAGE [19]. The results of qRNA revealed that the transcription level of some key genes during glycolysis (*FBA*, *PFK*, *PGK*), TCA pathway (*IDH*), glycogen metabolism (*GSY1*), gluconeogenesis (*PCK1*, *GPM*), and fatty acid metabolic processes (*FAS1*) were downregulated. Surfactin causes a significant change in the transcriptional profiling of *Candida albicans*, and there are 773 and 617 genes with at least a 1.5-fold increase or decrease in transcription, respectively [68]. Therefore, the growth inhibition of *F. foetens* was related to reduced expression of the enzymes involved in the basal metabolism of *F. foetens*. Surfactin-induced differential protein expression may have affected various fungal metabolic pathways.

Surfactin produced by *Bacillus* spp. induced an increase in reactive oxygen species (ROS), causing the death of *Magnaporthe grisea* [69]. Krishnan et al. presumed that surfactin induces ROS production in *F. moniliforme* causing protein and DNA damage [19]. However, no significant ROS changes were detected in our results (data not shown), and the *CPX* and *GSH*-*PX* genes involved in antioxidative defense were upregulated, indicating that ROS were not involved in inhibiting *F. foetens.* Surfactin exposure increased GSH production by *F. foetens*, which agreed with the upregulation of glutathione reductase-encoding genes and agrees with the results of Ágnes et al. [68]. GSH has free radical scavenging, anti-oxidation, and electrophile elimination activities [70]. The increase in the level of GSH in *F. foetens* was a response to surfactin.

DNA carries the genetic information of the entire organism and plays a significant role in the transcription, expression, growth, and development of organisms. DNA is also a target for exogenous substances to attack and cause changes in the body, leading to disease [71]. The lipopeptides, fengycin and iturin, bind with DNA, and the binding modes have been confirmed [39]. In our study, the interaction between surfactin and ff-DNA in groove binding mode was confirmed by UV-visible and fluorescence spectroscopy. Groove binders affect gene expression in vitro by inhibiting sequence selective binding of various transcription factors to DNA [72]. These effects may result in the expression or repression of downstream genes. Groove binding also results in structural distortion or damage to DNA that impedes replication and transcription by blocking the movement of helicases, topoisomerases, and polymerases [73]. We presumed that the interaction between surfactin and ff-DNA potentially altered replication or transcription, which was part of the mechanism by which *B. subtilis* SF1 inhibited *F. foetens*. However, the changes induced by the interaction between surfactin and ff-DNA and its importance in the antifungal effect need further study.

Surfactin mounts innate immunity in plants and thus decreases the invasive growth caused by fungal infection [12]. Inoculating *B. subtilis* SF1 after infection led to a significant decrease in the FW incidence rate (45.56%)*,* and healthy potato seedlings treated with *B. subtilis* SF1 appeared to grow vigorously, compared to untreated potato seedlings. Surfactin is essential for biofilm formation and surface motility of plant growth-promoting organisms [74,75]. Siderophore production and phosphate solubilization, both important for plant growth, were detected in *B. subtilis* SF1. *B. subtilis* SF1 also harbors many genes associated with root colonization, biofilm formation, swarming motility, and elicitation of plant basal defense. Thus, the alleviation of morbidity by *B. subtilis* SF1 was a combined effect of antifungal activity by surfactin and the secretion of various plant growth-promoting determinants.

## 5. Conclusions

The present study is the first to report that *F. foetens* infects potatoes and causes a large amount of FW disease. Surfactin was the main antifungal component produced by *B. subtilis* SF1. Surfactin inhibited mycelial growth, causing malformed mycelial morphology, cell leakage, differential protein expression, and GSH production. The underlying mechanisms of surfactin against *F. foetens* were presumed to be: (i) Surfactin altered cell membrane permeability, leading to lysis of cell contents and morphological malformation of the mycelia; (ii) surfactin caused differential protein expression and changes in cell metabolic pathways; (iii) surfactin bound to DNA in groove binding mode, impeding replication or transcription. Surfactin was a mixture of four homologs in our study. The presence of different types of surfactin in different proportions resulted in diverse antimicrobial activities. The antifungal activity of a single fraction or a mixture of different fractions of the surfactin produced by *B. subtilis* SF1 needs to be determined in further studies.

## Data Availability

The whole genome shotgun project for *Bacillus subtilis* SF1 has been deposited at DDBJ/ENA/Genbank under the accession JANYMH000000000. The sequences of ITS, EF-1α, and β-tubulin for *Fusarium foetens* have been submitted to NCBI with the accession number OM370930, OM370932, and OM370931, respectively. The authors state that the current study existing the necessary data for the conclusion and Supplementary Material will be fully exposed.

## Supplementary Materials

The following supporting information can be downloaded at: https://www.mdpi.com/article/10.3390/jof9030367/s1, Figure S1: Phylogenic tree inferred from the combined dataset of EF-1 α and β-tubulin based on the Neighbor-Joining method. *Fusarium foetens* isolate is the pathogen of potato wilt described in the study. The scale bar indicates genetic distance. Bootstrap values in 1000 replicates for major lineages at the nodes were shown as percentages; Figure S2: The effect of *B. subtilis* SF1 on the potato FW induced by *F. foetens*. (A) Seedlings inoculted with *F. foetens* cultured in pots put in the filed for 9 days. (B) Seedlings inoculted with *F. foetens* cultured in pots put in the filed for 2 weeks. (C) Healthy seedling. (D) Effect of *B. subtilis* SF1 on potato plants growth. The seedlings were transplanted into the pots with and without spores suspension of *B. subtilis* SF1 and then cultured in the greenhouse for 20 days. (T) the seedlings in the pots supplemented with 100 mL of spores suspension of *B. subtilis* SF1. (UT) the seedlings without any treatment; Figure S3: Effects of heat treatment on the antifungal activity of *B. subtilis* SF1 against *F. foetens*. The culture was incubated for 7 days at 25 °C. (A) Control: *F. foetens* on PDA. (B) *F. foetens* on PDA, replacing water by heated culture filtrate (105 °C for 10 min). (C) *F. foetens* on PDA supplemented with *B. subtilis* SF1 culture filtrate without heat treatment; Figure S4: LC-MS spectrometry analysis of surfactin produced by *B. subtilis* SF1. The positive-ion MS spectrum of surfactin yielded the expected ion of [M+Na]^+^ at *m*/*z* 1030 (S1), 1044 (S2), 1044(S3), and 1058 (S4), with molecular weights of 1007, 1021, 1021, and 1035 Da; Table S1: The genes encoding beta-glucanase, endo-1,4-beta-glucanase, and extracellular protease in the genome of *B. subtilis* SF1; Table S2: Representative genes involved in plant growth promotion in the genome of *B. subtilis* SF1; Table S3: The predicted secondary metabolites and the corresponding synthesis gene clusters in the genome of *B. subtilis* SF1.

## References

[1] Devaux A., Kromann P., Ortiz O. (2014). Potatoes for sustainable global food security. Potato Res..

[2] Tiwari R.K., Kumar R., Sharma S., Sagar V., Aggarwal R., Naga K.C., Kumar M. (2020). Potato dry rot disease: Current status, pathogenomics and management. 3 Biotech.

[3] Oerke E.C. (2005). Crop losses to pests. J. Agric. Sci..

[4] Larkin R.P., Halloran J.M. (2014). Management effects of disease-suppressive rotation crops on potato yield and soilborne disease and their economic implications in potato production. Am. J. Potato Res..

[5] Xia S.Y., Niu Z.M., Li Q.Q., Zhang L.J., Sheng W.M. (2022). Research progress and control measures of *Fusarium* wilt of potato. Heilongjiang Agric. Sci..

[6] Nxumalo N.N. (2013). Occurrence, Identification and a Potential Management Strategy of Fusarium Species Causing wilt of Potatoes in South Africa. Univ. Pretoria.

[7] Mejdoub-Trabelsi B., Touihri S., Ammar N., Riahi A., Daami-Remadi M. (2019). Effect of chitosan for the control of potato diseases caused by *Fusarium* species. J. Phytopathol..

[8] Paul N.C., Park W., Lee S., Chung M.N., Lee H.U., Yang J.W. (2020). Occurrence of sweetpotato (*Ipomoea batatas*) wilt and surface rot disease and determining resistance of selected varieties to the pathogen in Korea. Plants.

[9] Ayed F., Daami-Remadi M., Jabnoun-Khiareddine H., Hibar K., Mahjoub M.E. (2006). Evaluation of fungicides for control of *Fusarium* wilt of potato. Plant Pathol. J..

[10] Ayed F., Daami-Remadi M., Jabnoun-Khiareddine H., Hibar K., Mahjoub M.E. (2006). Effect of potato cultivars on incidence of *Fusaium oxysporum* f. sp. tuberosi and its transmission to progeny tubers. Agron. J..

[11] Khedher S.B., Mejdoub-Trabelsi B., Tounsi S. (2020). Biological potential of *Bacillus subtilis* V26 for the control of *Fusarium* wilt and tuber dry rot on potato caused by *Fusarium* species and the promotion of plant growth. Biol. Control.

[12] Gouda S., Kerry R.G., Das G., Paramithiotis S., Shin H.S., Patra J.K. (2018). Revitalization of plant growth promoting rhizobacteria for sustainable development in agriculture. Microbiol. Res..

[13] Arrebola E., Sivakumar D., Bacigalupo R., Korsten L. (2010). Combined application of antagonist *Bacillus amyloliquefaciens* and essential oils for the control of peach postharvest diseases. Crop Prot..

[14] Chen X., Lu Y., Shan M., Zhao H., Lu Z., Lu Y. (2022). A mini-review: Mechanism of antimicrobial action and application of surfactin. World J. Microbiol. Biotechnol..

[15] Dimkić I., Živković S., Berić T., Ivanović Ž., Gavrilović V., Stanković S., Fira D. (2013). Characterization and evaluation of two *Bacillus* strains, SS-12.6 and SS-13.1, as potential agents for the control of phytopathogenic bacteria and fungi. Biol. Control.

[16] Yuan B., Xu P.Y., Zhang Y.J., Wang P.P., Yu H., Jiang J.H. (2014). Synthesis of biocontrol macromolecules by derivative of chitosan with surfactin and antifungal evaluation. Int. J. Biol. Macromol..

[17] Jiang J., Gao L., Bie X., Lu Z., Liu H., Zhang C., Zhao H. (2016). Identification of novel surfactin derivatives from NRPS modification of *Bacillus subtilis* and its antifungal activity against *Fusarium moniliforme*. BMC Microbiol..

[18] Agarwal M., Dheeman S., Dubey R.C., Kumar P., Maheshwari D.K., Bajpai V.K. (2017). Differential antagonistic responses of *Bacillus pumilus* MSUA3 against *Rhizoctonia solani* and *Fusarium oxysporum* causing fungal diseases in *Fagopyrum esculentum* moench. Microbiol. Res..

[19] Krishnan N., Velramar B., Velu R.K. (2019). Investigation of antifungal activity of surfactin against mycotoxigenic phytopathogenic fungus *Fusarium moniliforme* and its impact in seed germination and mycotoxicosis. Pestic. Biochem. Physiol..

[20] Elmer W.H., Vossbrinck C., Geiser D.M. (2004). First report of a wilt disease of *Hiemalis begonias* caused by *Fusarium foetens* in the United States. Plant Dis..

[21] Schroers H.J., Baayen R.P., Meffert J.P., de Gruyter J., Hooftman M., O’Donnell K. (2004). *Fusarium foetens*, a new species pathogenic to begonia elatior hybrids (*Begonia × hiemalis*) and the sister taxon of the *Fusarium oxysporum* species complex. Mycologia.

[22] Tian X.L., Dixon M., Zheng Y. (2010). First report of Hiemalis begonias wilt disease caused by *Fusarium foetens* in Canada. Plant Dis..

[23] Saurat C., Fourrier C., Wilson V., Casset C., Ioos R. (2013). First report of begonia elatior wilt disease caused by *Fusarium foetens* in France. Plant Dis..

[24] Lamprecht S.C., Tewoldemedhin Y.T. (2017). *Fusarium* species associated with damping-off of rooibos seedlings and the potential of compost as soil amendment for disease suppression. S. Afr. J. Bot..

[25] Amobonye A., Bhagwat P., Ranjith D., Mohanlall V., Pillai S. (2021). Characterisation, pathogenicity and hydrolytic enzyme profiling of selected *Fusarium* species and their inhibition by novel coumarins. Arch. Microbiol..

[26] Xu L.L., Zhang Y.J., Guo L.Z., Liu L. (2021). First report of *Colletotrichum gloeosporioides* causing leaf spot on *Cyclobalanopsis glauca* in China. Plant Dis..

[27] O’Donnell K., Cigelnik E. (1997). Two divergent intragenomic rDNA ITS2 types within a monophyletic lineage of the fungus *Fusarium* are nonorthologous. Mol. Phylogenet. Evol..

[28] Baayen R.P., O’Donnell K., Bonants P.J.M., Cigelnik E., Kroon L.P.N.M., Roebroeck E.J.A., Waalwijk C. (2000). Gene genealogies and AFLP analyses in the *Fusarium oxysporum* complex identify monophyletic and nonmonophyletic formae speciales causing wilt and rot disease. Phytopathology.

[29] Sarwar A., Hassan M.N., Imran M., Iqbal M., Majeed S., Brader G., Hafeez F.Y. (2018). Biocontrol activity of surfactin A purified from *Bacillus* NH-100 and NH-217 against rice bakanae disease. Microbiol. Res..

[30] Li S.S. (2017). Field Application of *Bacillus amyloliquefaciens* S3-1 and Technology Optimization of Surfactin Fermentation. Master’s Thesis.

[31] Yokota K., Hayakawa H. (2015). Impact of antimicrobial lipopeptides from *Bacillus* sp. on suppression of fusarium yellows of tatsoi. Microbes Environ..

[32] Teather R.M., Wood P.J. (1982). Use of congo red-polysaccharide interactions in enumeration and characterization of cellulolytic bacteria from the bovine rumen. Appl. Environ. Microbiol..

[33] Dinesh R., Anandaraj M., Kumar A., Bini Y.K., Subila K.P., Aravind R. (2015). Isolation, characterization, and evaluation of multi-trait plant growth promoting rhizobacteria for their growth promoting and disease suppressing effects on ginger. Microbiol. Res..

[34] Frey-Klett P., Chavatte M., Clausse M.L., Courrier S., Roux C.L., Raaijmakers J., Garbaye J. (2004). Ectomycorrhizal symbiosis affects functional diversity of rhizosphere fluorescent pseudomonads. New Phytol..

[35] Schwyn B., Neilands J.B. (1987). Universal chemical assay for the detection and determination of siderophores. Anal. Biochem..

[36] Bric J.M., Bostock R.M., Silverstone S.E. (1991). Rapid in situ assay for indoleacetic acid production by bacteria immobilized on a nitrocellulose membrane. Appl. Environ. Microbiol..

[37] Luo C., Liu X., Zhou H., Wang X., Chen Z. (2015). Nonribosomal peptide synthase gene clusters for lipopeptide biosynthesis in *Bacillus subtilis* 916 and their phenotypic functions. Appl. Environ. Microbiol..

[38] Jones R.N., Ballow C.H., Biedenbach D.J. (2001). Multi-laboratory assessment of the linezolid spectrum of activity using the Kirby-Bauer disk diffusion method: Report of the Zyvox^®^ Antimicrobial Potency Study (ZAPS) in the United States. Diagn. Microbiol. Infect. Dis..

[39] Liu Y., Lu J., Sun J., Lu F., Bie X., Lu Z. (2019). Membrane disruption and DNA binding of *Fusarium graminearum* cell induced by C16-Fengycin A produced by *Bacillus amyloliquefaciens*. Food Control.

[40] Ahmadi F., Alizadeh A.A., Bakhshandeh-Saraskanrood F., Jafari B., Khodadadian M. (2010). Experimental and computational approach to the rational monitoring of hydrogen-bonding interaction of 2-Imidazolidinethione with DNA and guanine. Food Chem. Toxicol..

[41] Oguzcan E., Koksal Z., Taskin-Tok T., Uzgoren-Baran A., Akbay N. (2022). Spectroscopic and molecular modeling methods to investigate the interaction between psycho-stimulant modafinil and calf thymus dna using ethidium bromide as a fluorescence probe. Spectrochim. Acta Part A.

[42] Rehman S.U., Sarwar T., Husain M.A., Ishqi H.M., Tabish M. (2015). Studying non-covalent drug-DNA interactions. Arch. Biochem. Biophys..

[43] Cao Y., He X. (1998). Studies of interaction between Safranine T and double helix DNA by spectral methods. Spectrochim. Acta Part A.

[44] Marty R., N’soukpoe-Kossi C.N., Charbonneau D., Weinert C.M., Kreplak L., Tajmir-Riahi H.A. (2008). Structural analysis of DNA complexation with cationic lipids. Nucleic Acids Res..

[45] Ni Y., Wang Y., Kokot S. (2011). Study of the interaction between 10-hydroxycamptothecine and DNA with the use of ethidium bromide dye as a fluorescence probe. Sens. Actuators B.

[46] Howe-Grant M., Wu K.C., Bauer W.R., Lippard S.J. (1976). Binding of platinum and palladium metallointercalation reagents and antitumor drugs to closed and open DNAs. Biochemistry.

[47] Sehlstedt U., Kim S.K., Carter P., Goodisman J., Vollano J.F., Norden B., Dabrowiak J.C. (1994). Interaction of cationic porphyrins with DNA. Biochemistry.

[48] Tsan P., Volpon L., Besson F., Lancelin J.M. (2007). Structure and dynamics of surfactin studied by NMR in micellar media. J. Am. Chem. Soc..

[49] Bojanowski A., Avis T.J., Pelletier S., Tweddell R.J. (2013). Management of potato dry rot. Postharvest Biol. Technol..

[50] Chung S., Kong H., Buyer J.S., Lakshman D.K., Lydon J., Kim S.D., Roberts D.P. (2008). Isolation and partial characterization of *Bacillus subtilis* ME488 for suppression of soilborne pathogens of cucumber and pepper. Appl. Microbiol. Biotechnol..

[51] Yu X., Ai C., Xin L., Zhou G. (2011). The siderophore-producing bacterium, *Bacillus subtilis* CAS15, has a biocontrol effect on *Fusarium* wilt and promotes the growth of pepper. Eur. J. Soil Biol..

[52] Zhang L., Khabbaz S.E., Wang A., Li H., Abbasi P.A. (2015). Detection and characterization of broad-spectrum antipathogen activity of novel rhizobacterial isolates and suppression of *Fusarium* crown and root rot disease of tomato. J. Appl. Microbiol..

[53] Zhao Q., Ran W., Wang H., Li X., Shen Q., Shen S., Xu Y. (2012). Biocontrol of *Fusarium* wilt disease in muskmelon with *Bacillus subtilis* Y-IVI. Bio. Control.

[54] De Weerdt M., Zijlstra C., Van Brouwershaven I.R., Van Leeuwen G.C.M., De Gruyter J., Kox L.F.F. (2006). Molecular detection of *Fusarium foetens* in *Begonia*. J. Phytopathol..

[55] Chang W.T., Chen M.L., Wang S.L. (2010). An antifungal chitinase produced by *Bacillus subtilis* using chitin waste as a carbon source. World J. Microbiol. Biotechnol..

[56] Luo Y., Sun L., Zhu Z., Ran W., Shen Q. (2013). Identification and characterization of an anti-fungi *Fusarium oxysporum* f. sp. cucumerium protease from the *Bacillus subtilis* strain N7. J. Microbiol..

[57] Podile A.R., Prakash A.P. (1996). Lysis and biological control of *Aspergillus niger* by *Bacillus subtilis* AF 1. Can. J. Microbiol..

[58] Aktuganov G.E., Galimzyanova N.F., Melent’ev A.I., Kuz’mina L.Y. (2007). Extracellular hydrolases of strain *Bacillus* sp. 739 and their involvement in the lysis of micromycete cell walls. Microbiology.

[59] Siahmoshteh F., Hamidi-Esfahani Z., Spadaro D., Shams-Ghahfarokhi M., Razzaghi-Abyaneh M. (2018). Unraveling the mode of antifungal action of *Bacillus subtilis* and *Bacillus amyloliquefaciens* as potential biocontrol agents against aflatoxigenic Aspergillus parasiticus. Food Control.

[60] Hu L.B., Shi Z.Q., Zhang T., Yang Z.M. (2007). Fengycin antibiotics isolated from B-FS01 culture inhibit the growth of *Fusarium moniliforme* Sheldon ATCC 38932. FEMS Microbiol. Lett..

[61] Lin L.Z., Zheng Q.W., Wei T., Zhang Z.Q., Zhao C.F., Zhong H., Guo L.Q. (2020). Isolation and characterization of fengycins produced by *Bacillus amyloliquefaciens* JFL21 and its broad-spectrum antimicrobial potential against multidrug-resistant foodborne pathogens. Front. Microbiol..

[62] Zhao Z., Wang Q., Wang K., Brian K., Liu C., Gu Y. (2010). Study of the antifungal activity of *Bacillus vallismortis* ZZ185 in vitro and identification of its antifungal components. Bioresour. Technol..

[63] Sheppard J.D., Jumarie C., Cooper D.G., Laprade R. (1991). Ionic channels induced by surfactin in planar lipid bilayer membranes. Biochim. Biophys. Acta Biomembr..

[64] Carrillo C., Teruel J.A., Aranda F.J., Ortiz A. (2003). Molecular mechanism of membrane permeabilization by the peptide antibiotic surfactin. Biochim. Biophys. Acta Biomembr..

[65] Zhu J., Tan T., Shen A., Yang X., Yu Y., Gao C., Zeng L. (2020). Biocontrol potential of *Bacillus subtilis* IBFCBF-4 against *Fusarium* wilt of watermelon. J. Plant Pathol..

[66] Backhouse D., Stewart A. (1989). Interactions between *Bacillus* species and sclerotia of *Sclerotium cepivorum*. Soil Biol. Biochem..

[67] Benoit I., van den Esker M.H., Patyshakuliyeva A., Mattern D.J., Blei F., Zhou M., Kovács Á.T. (2014). *Bacillus subtilis* attachment to *Aspergillus nigerhyphae* results in mutually altered metabolism. Environ. Microbiol..

[68] Ágnes J., Fruzsina K., Noémi B., Zoltán T., Ágota R., Zsófi S., Kinga C., Csaba N.K., Dániel N., Ildikó B. (2022). Physiological and transcriptional profiling of surfactin exerted antifungal effect against *Candida albicans*. Biomed. Pharmacother..

[69] Wu S., Liu G., Zhou S., Sha Z., Sun C. (2019). Characterization of antifungal lipopeptide biosurfactants produced by marine bacterium *Bacillus* sp. CS30. Mar. Drugs.

[70] Pócsi I., Prade R.A., Penninckx M.J. (2004). Glutathione, altruistic metabolite in fungi. Adv. Microb. Physiol..

[71] Boer D.R., Canals A., Coll M. (2009). DNA-binding drugs caught in action: The latest 3D pictures of drug-DNA complexes. Dalton Trans..

[72] Turner P.R., Denny W.A. (1996). The mutagenic properties of DNA minor-groove binding ligands. Microbiol. Res..

[73] Nelson S.M., Ferguson L.R., Denny W.A. (2007). Non-covalent ligand/DNA interactions: Minor groove binding agents. Mutat. Res..

[74] Cuellar-Gaviria T.Z., González-Jaramillo L.M., Villegas-Escobar V. (2021). Role of *Bacillus tequilensis* EA-CB0015 cells and lipopeptides in the biological control of black Sigatoka disease. Biol. Control.

[75] Dong L.H., Wang P.P., Zhao W.S.S., Su Z.H., Zhang X.Y., Lu X.Y., Li S.Z., Ma P., Guo Q.G. (2022). Surfactin and fengycin contribute differentially to the biological activity of *Bacillus subtilis* NCD-2 against cotton verticillium wilt. Biol. Control.

