# Supplementary material for: Mechanisms of Surfactin from Bacillus subtilis SF1 against Fusarium foetens: A Novel Pathogen Inducing Potato Wilt
Source: J Fungi (Basel). 2023 Mar 17;9(3):367. doi: 10.3390/jof9030367 (PMC10056554; doi:10.3390/jof9030367)
Supplement: Supplementary file 1 [file jof-09-00367-s001.zip › jof-2261367-supplementary.pdf]

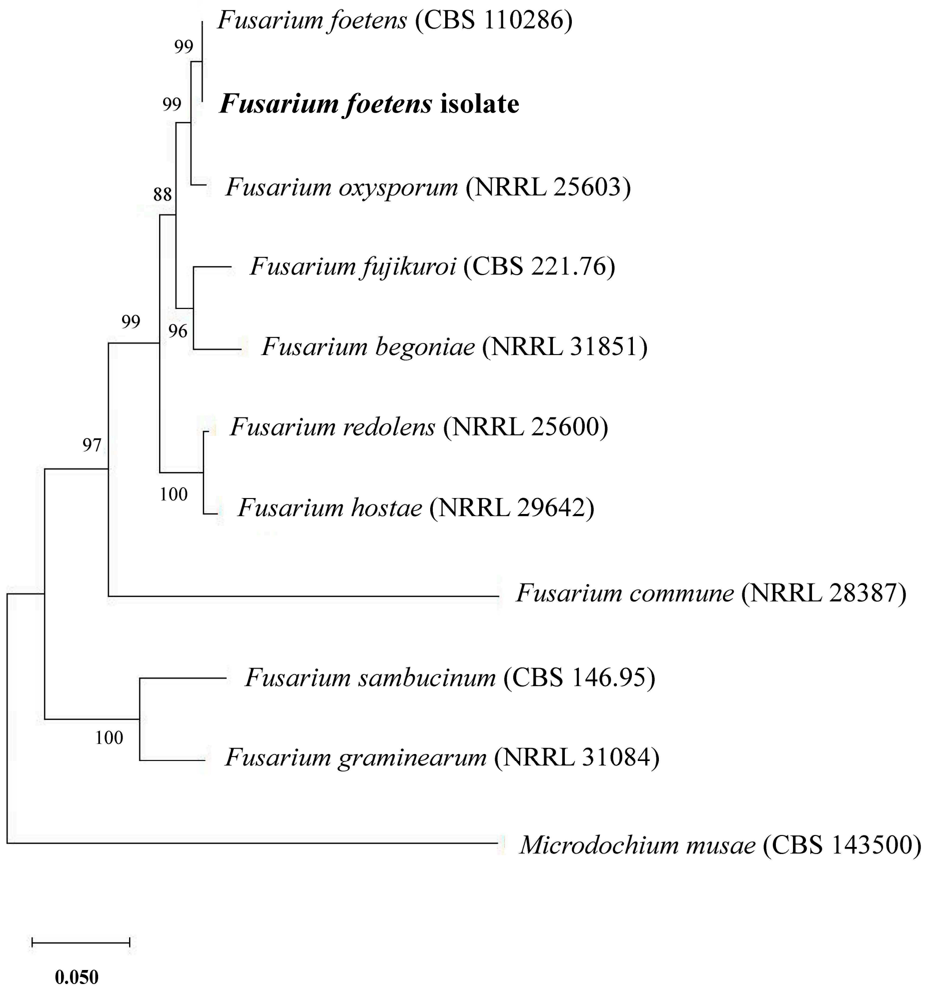

Figure S1. Phylogenic tree inferred from the combined dataset of EF-1 α and β-tubulin based on the Neighbor-Joining method. *Fusarium foetens* isolate is the pathogen of potato wilt described in the study. The scale bar indicates genetic distance. Bootstrap values in 1000 replicates for major lineages at the nodes were shown as percentages.

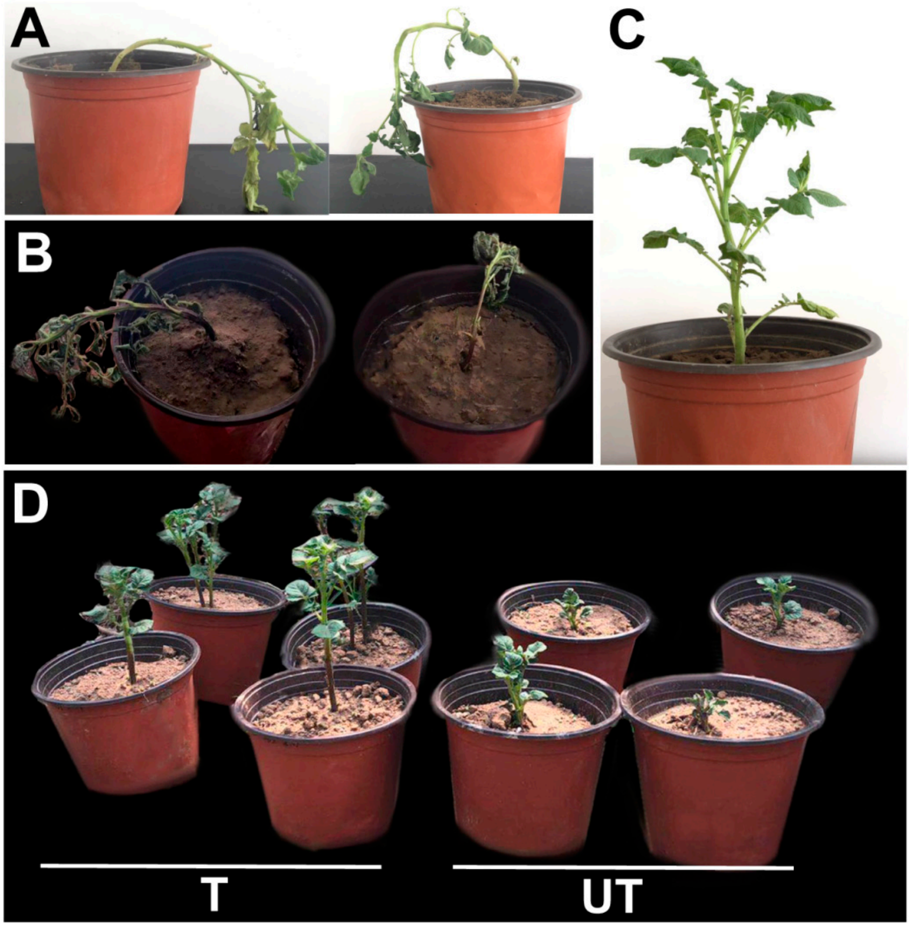

Figure S2. The effect of *B. subtilis* SF1 on the potato FW induced by *F. foetens.* **(A)** Seedlings inoculted with *F. foetens* cultured in pots put in the filed for 9 days. **(B)** Seedlings inoculted with *F. foetens* cultured in pots put in the filed for 2 weeks. **(C)** Healthy seedling. **(D)** Effect of *B. subtilis* SF1 on potato plants growth. The seedlings were transplanted into the pots with and without spores suspension of *B. subtilis* SF1 and then cultured in the greenhouse for 20 days. **(T)** the seedlings in the pots supplemented with 100 ml of spores suspension of *B. subtilis* SF1. **(UT)** the seedlings without any treatment.

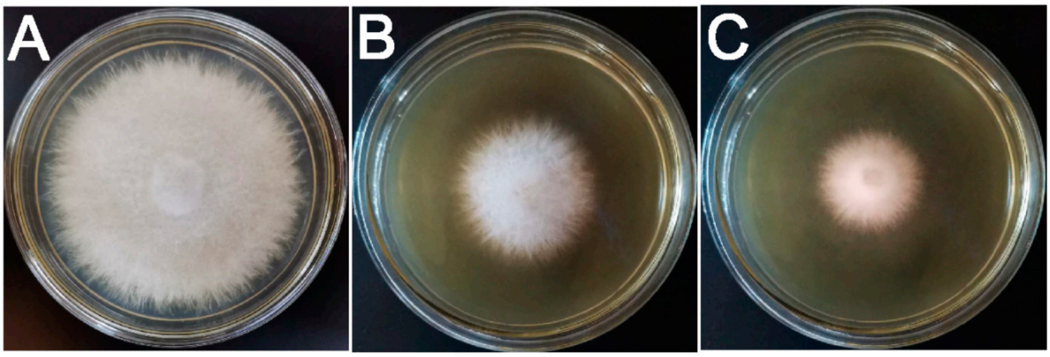

Figure S3. Effects of heat treatment on the antifungal activity of *B. subtilis* SF1 against *F. foetens*. The culture was incubated for 7 days at 25 °C. **(A)** Control: *F. foetens* on PDA. **(B)** *F. foetens* on PDA, replacing water by heated culture filtrate (105 °C for 10 min). **(C)** *F. foetens* on PDA supplemented with *B. subtilis* SF1 culture filtrate without heat treatment.

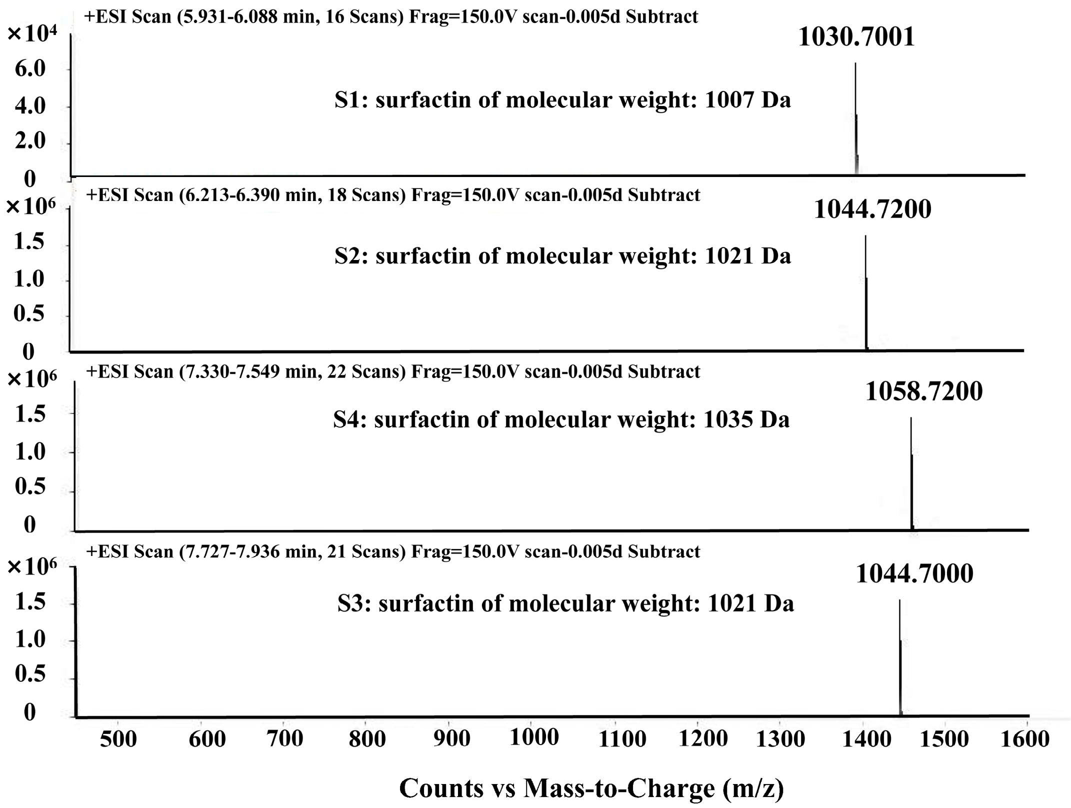

Figure S4. LC-MS spectrometry analysis of surfactin produced by *B. subtilis* SF1. The positive-ion MS spectrum of surfactin yielded the expected ion of $[M+Na]^+$ at m/z 1030 (S1), 1044 (S2), 1044(S3), and 1058 (S4), with molecular weights of 1007, 1021, 1021, and 1035 Da.

Table S1. The genes encoding beta-glucanase, endo-1,4-beta-glucanase, and extracellular protease in the genome of *B. subtilis* SF1.

Gene	Protein	Position	Description
eglS	endo-1,4-beta glucanase EglS	988,650-990,149	Hydrolytic activity against carboxymethycellulose
bglS	beta-glucanase	835,782-836,510	Hydrolyze beta-glucans such as lichenan or barley beta-glucan
nprE	neutral metalloprotease NprE	586,981-588,546	Neutral metalloprotease
epr	protease Epr	762,622-764,559	Serine protease
vpr	serine protease Vpr	728,750-731,170	Fibrinolytic enzyme, a serine protease of the subtilisin family
bpr	bacillopeptidase F	646,231-650,532	Serine proteinase with esterolytic activity
wprA	cell wall-associated protease WprA	203,965-206,649	Subtilisin protease family

Table S2. Representative genes involved in plant growth promotion in the genome of *B. subtilis* SF1.

Gene	Position	Description
spo0A	1,433,063 to 1,433,866	Biofilm formation
sigW	424,349 to 424,912	Biofilm formation
sinR	1,467,687 to 1,468,028	Biofilm formation
sinI	1,467,659 to 1,467,486	Biofilm formation
abrB	45,441 to 45,725	Biofilm formation
resE	1,332,210 to 1,330,441	Biofilm formation
lytS	1,887,433 to 1,885,652	Biofilm formation
ybdK	397,849 to 396,863	Biofilm formation
ycbA	355,282 to 356,634	Biofilm formation
EpsA to O	332,476 to 348,216	Necessary for biofilm formation
yqxM to	1,468,121 to 1,470,287	Essential for biofilm formation
tasA	354,374 to 355,795	Root adhesion
sacB	1,453,155 to 1,453,712	Essential for swarming motility
efp	764,229 to 764,732	Essential for swarming motility
swrB	441,011 to 441,346	Essential for swarming motility
swrA	204,256 to 207,414	Self-resistance against surfactin
swrC	74,898 to 77,141	Regulator of surfactin production
comP	218,867 to 245,015	Necessary for surface motility and biofifilm
srfABCD	213,097 to 213,744	formation
yczE	214,523 to 213,849	Necessary for surface motility and biofifilm
sfp	127,632 to 130,324	formation
escABC	615,368 to 615,817	Necessary for surface motility and biofifilm

ylbF	821,321 to 821,752	formation
ymcA	26,810 to 28,000	Protein secretion to extracellular matrix
tuf	452,303 to 453,799	Control of community development
fliD	454,379 to 455,293	Control of community development
hag	457,637 to 459,160	Elicitation of plant basal defence
flgK	468,046 to 478,310	Elicitation of plant basal defence
tuaA to		Elicitation of plant basal defence
tagO		Elicitation of plant basal defence
		Elicitation of plant basal defence

Table S3. The predicted secondary metabolites and the corresponding synthesis gene clusters in the genome of *B. subtilis* SF1.

Metabolite	Gene cluster	Size	Position	Bioactive spectrum
Surfactin	*srfABCD, ycxA, sfp, yczE*	31.9 Kb	2,736,952 to 2,768,870	Mycoplasmra, fungi, virus and tumor
Pilpastatin	*ppsABCDE*	37.7 Kb	1,429,921 to 2,768,870	Bacteria and fungi
Bacilysin	*bacABCDEFG*	6.7 Kb	3,657,315 to 3,664,002	Bacteria and *Candida albicans*
Bacillaene	*pksABCFGHIJKLM NRS,acpK*	77.9 Kb	1,250,587 to 1,328,470	Bacteria
Bacillibacti n	*dhbBCEF*	2.6 Kb	3,076,306 to 3,078,940	Microbial competitors
Sublitolin	*albABCDEFG*	6.7 Kb	3,625,712 to 3,632,518	Bacteria
